# Linking health and education data to plan and evaluate services for children

**DOI:** 10.1136/archdischild-2016-311656

**Published:** 2017-01-27

**Authors:** Johnny Downs, Ruth Gilbert, Richard D Hayes, Matthew Hotopf, Tamsin Ford

**Affiliations:** 1 Department of Psychological Medicine, Institute of Psychiatry, Psychology and Neuroscience, King's College London, London, UK; 2 NIHR Biomedical Research Centre for Mental Health at the South London and Maudsley NHS Foundation Trust, London, UK; 3 Farr Institute of Health Informatics Research London, London, UK; 4 Children's Policy Research Unit, UCL Institute of Child Health, London, UK; 5 Child Mental Health Research Group, University of Exeter Medical School, Exeter, UK

**Keywords:** Health services research, Outcomes research, Information Technology, Child Psychiatry, School Health

## Introduction

Linkage of routinely collected data from public services has the potential to improve how local health, education and social care are delivered to children. All mental health services, hospital-based child health services, schools and child protection services which serve the same local area could be more efficient if the design, monitoring, targeting and integration of services were based on data. Health services need evidence from the populations that they serve to plan care and know whether they are meeting children's needs, duplicating effort or allowing some children to fall through the net. In this paper, we describe how the Clinical Record Interactive Search (CRIS) programme has joined up data from health, education and social services for children living in four local authorities in South London to create two datasets: one linking hospital to children's mental health services and the second linking mental health data to education data. We describe these resources, give examples of how they are being used to improve services and discuss what is needed to implement this approach more widely across the UK.

## What data are available?

Across England, all National Health Service (NHS) and state education services for children routinely generate administrative data, but few areas have managed to join these data systematically to evaluate how services could better serve their populations. Details of every NHS hospital inpatient admission, emergency department and outpatient contact are centrally collated by NHS Digital.^1^ Demographic and socioeconomic data on every child in state education are submitted by all state-maintained schools to the Department of Education, along with the information on school attendance, attainment, exclusion, child protection involvement and special educational needs.^2^ Centrally collected child mental health data have yet to become available, but nearly all local services collect these data within their electronic health record systems.^3^ A big challenge is meeting the technical and governance requirements that safeguard sensitive child data, and also permit the linkage across public service data resources. This challenge has been addressed by the National Institute for Health Research (NIHR) Biomedical Research Centre at the Maudsley, and there is the potential to extend our approach to other sites.

Ten years ago, the Maudsley NIHR Biomedical Research Centre set up CRIS. CRIS is a secure data repository for NHS mental health services covering four local authorities. CRIS developed a process to anonymise electronic health records for service evaluation and clinical research. The system operates strict governance controls with external service user led oversight.^4^ ^5^ CRIS retains patient identifiers, such as NHS number, separately from patients' mental health records, which are anonymised.

CRIS has linked mental health data to education and hospital data. It took 3 years, from first application, to obtain permissions to do this from the Health Research Authority, NHS Digital and the Department for Education. The linkage process itself involved CRIS sending patient identifiers (names, dates of birth and postcodes) without any mental health information to NHS Digital and to the Department for Education, where the identifiers were linked, and data from education and hospitals were deidentified and returned to CRIS. The CRIS secure environment now holds two linked datasets: education data linked to mental health data, and a second dataset containing mental health and hospital data. These datasets are kept separately, with all identifiers (names and NHS or pupil ID numbers) removed.

The CRIS system covers all NHS mental health services for four local authorities, which serve a population of 1.25 million people. Patients using mental health services are made aware of how their data are used through notices in clinics, websites and regular public engagement events. Although patients are not asked for consent to use their data for service evaluation or research, they are able to opt out. Only three individuals have asked to opt out of CRIS in 6 years. In 2014, the CRIS system was extended to four more mental health trusts (in 16 local authorities) and could be extended beyond mental health to other services.^6^

## Using linked data from schools and mental health services

### The population

**Figure 1:**
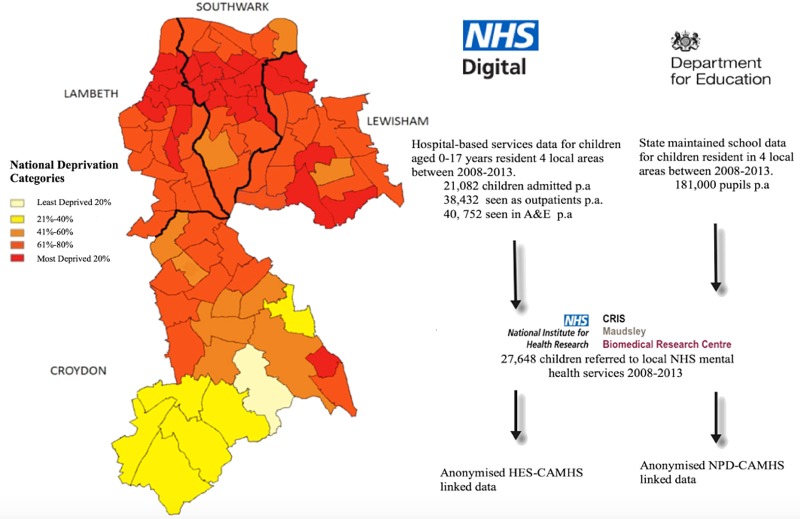
Linked data resources to provide an anonymised multiagency dataset covering child and adolescent mental health services, hospital attendances, education services and social service activity in South London. CRIS, Clinical Record Interactive Search; NHS, National Health Service.

The linked schools and mental health dataset capture data for approximately 160 000–190 000 children each year from 2007 to 2013. To be included, children need to be aged between 4 and 16 years (see figure 1 for population numbers) and be resident in Southwark, Lambeth, Lewisham or Croydon. These areas are culturally and economically diverse, representing both outer and inner London regions. The catchment population has substantially higher proportions of families from black minority ethnic groups and/or born outside UK compared with the rest of London and England. Highest and lowest socioeconomic groups are overly represented compared with England, with higher rates of unemployment and also higher levels of education.^7^ Linkage with the national pupil dataset (NPD) means that information on education is still captured for those attending state school outside the local catchment area, and for those who move in or out of the area. Some of the population are not routinely captured. Children attending independent (meaning private) primary schools are not represented (∼5% of the population aged under 12).^8^ Children attending independent secondary schools are included when they sit any national examinations (eg, General Certificate of Secondary Education (GCSE) or A level).

### What can be measured?

Alongside sociodemographic characteristics, the national pupil dataset provides rich information on childhood development.^2^ It tracks indicators of cognitive ability via routine teacher-based assessment of language and numerical ability as children start school, and then via standardised academic assessments in mid and late childhood. It captures indicators of special educational needs such as physical problems, including deafness and visual impairment; emotional and behavioural problems; and autism spectrum disorder and learning disability. The dataset also captures episodes of children being excluded from school and indicators of absenteeism. Children's social care data have also been linked, which include social service referrals and investigations, including details of children who are placed into out-of-home care.

The CRIS system enables researchers to access electronic mental health record data for approved studies.^5^ ^9^ Data available for research include structured information (eg, data entered by clinicians from drop-down lists), such as past and present International statistical classification of diseases, 10th revision psychiatric diagnoses, appointments attended and routine outcome measures (eg, Strength and Difficulties Questionnaires),^10^ and risk assessment details including risk of self-harm, self-injury and aggression to others.^11^ Natural language processing software is used to enhance these data by extracting information predominately found in clinical progress notes and correspondence that might include more detail about family mental health problems, substance misuse, pharmacotherapy and symptoms.^9^

## How can school and mental health data be used to improve services?

The linked school and mental health data have many potential applications. They can provide detailed information on patient pathways and the extent of inequalities to services. This information can be used to flag gaps in existing healthcare provision and direct where new services are needed. National and local surveys have provided consistent evidence that timely access to services varies by social status and area of residence.^12^ Data suggest that young people at high risk for mental health problems, looked after children and care leavers, those at risk of social exclusion or who have experienced abuse, or those with long-term physical health conditions, are the ‘hardest to reach’ and more likely to receive insufficient or fragmented care.^13^ Because local areas have considerable flexibility in how they commission child mental health services, there is a risk that ‘hard to reach’ groups are least likely to receive services. Mental health–school linked data can help understand which children receive support among socially vulnerable groups in each local area. Using data in this way to map service provision is particularly pertinent for integrated child health programmes which aim to tackle the potential inefficiencies and inequalities of current condition-specific pathways.^14^ At present, local areas have very limited information on how mental health resources are accessed by vulnerable children, which include looked after children, those with a history of social services contact, prolonged absences from school,^15^ permanent exclusions^16^ and with complex education needs.^17^ Using these linked data, we can gain a clearer picture of how well education and mental services overlap to address emotional and behavioural difficulties, and the shared awareness of special educational needs across both services.

**Figure 2:**
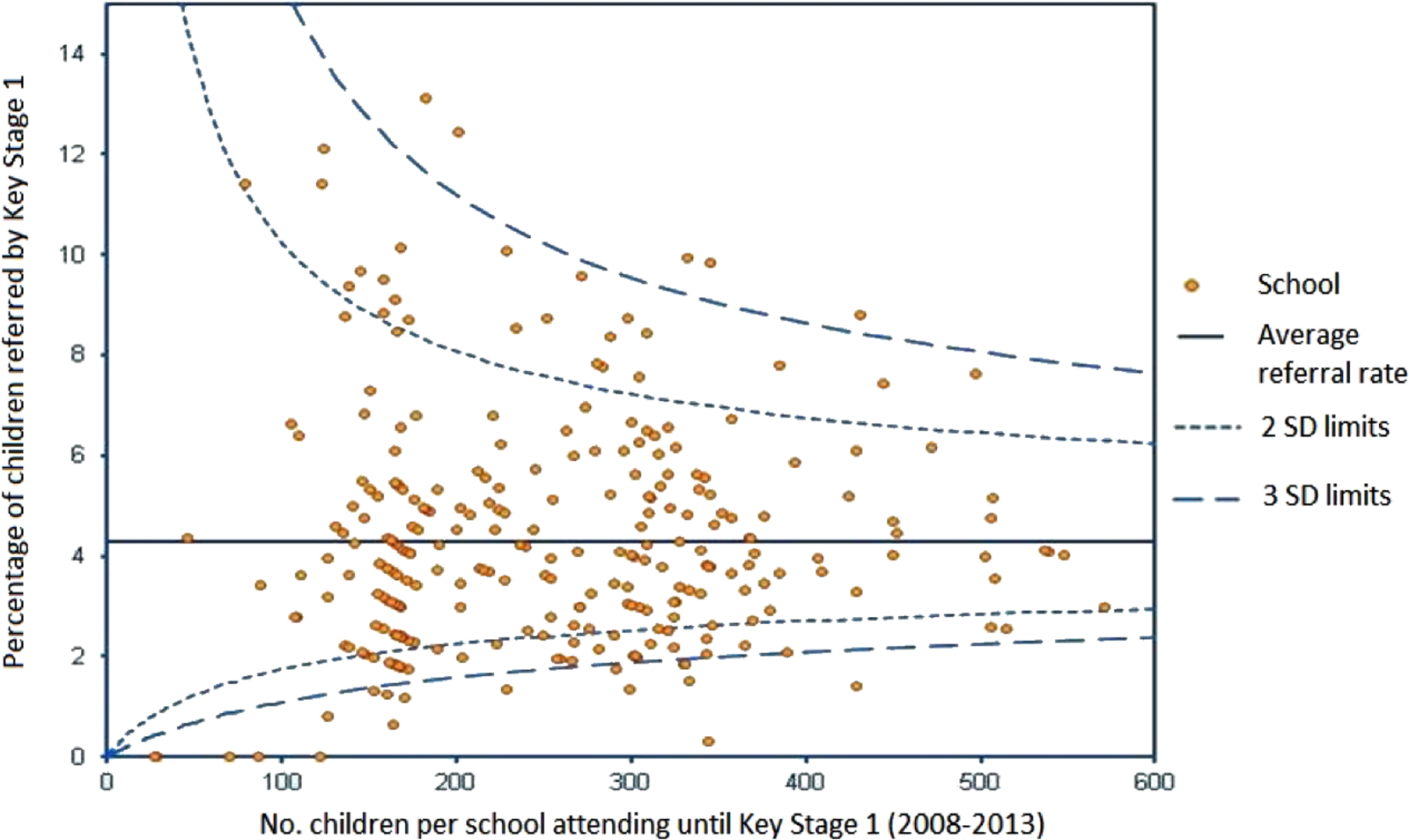
Plot showing referral rates to Child and Adolescent Mental Health Services for each school by Key Stage 1 (infant school) as a function of the pupils enrolled between 2008 and 2013. The average referral rate is 4.3% (shown as a horizontal line). Control limits are also plotted above and below this mean.

Linkage of schools’ data to mental health services also offers opportunities for targeting school-based prevention strategies. For example, the funnel plot in figure 2 shows variation between mainstream schools in referrals to child and adolescent mental health services in the four local areas for children aged <8 years. Outliers on the funnel plots are of particular interest and warrant further exploration: very high rates could reflect high levels of population need and/or school-wide difficulties in managing emotional and behavioural problems, or conversely, very low referrals to mental health services may reflect excellent in-school support and provide a model of good practice. Using similar techniques, the data can be used to examine whether potentially more ‘contagious’ adolescent mental health problems such as eating disorders, self-harm or suicidal behaviours cluster within schools. Findings can then be used to prioritise schools for preventive strategies. There is also a need for research to examine the associations between educational achievement, self-harm presenting to mental health services and the potential impact of school-based interventions, as almost no research has been conducted on this topic in the UK.^18^

## Using linked hospital–mental health service data to inform services

There are a number of policy-relevant research areas that can benefit from using linked mental health and hospital administrative data. One example is the evaluation of policy initiatives to improve the quality of crisis care for young people.^19^ There are approximately 200 000 episodes of self-harm that present to emergency services each year in the UK, with the highest rates among adolescents and young adults.^20^ ^21^ Between 25% and 50% of adolescents presenting to emergency care with self-harm do not attend any follow-up mental health support.^22–24^ Emergency departments have an important influence on future engagement with treatment.^25^ By adapting an approach developed in adult populations,^26^ we can track temporal shifts in the rates of emergency department attendances for self-harm or suicidal behaviour for the 40 752 children and adolescents seen each year from the four local authorities served by CRIS. We can assess whether practice changes in emergency departments result in reduced rates of attendance for self-harm in the long term.

Another example is the use of linked hospital–mental health data to follow up children hospitalised with long-term conditions to investigate their use of mental health services and psychiatric comorbidity. We can evaluate the types of patients who receive mental healthcare, when and which factors are associated with treatment gaps and/or reliance on emergency care and unplanned admissions. These studies can provide information on whether systems of care need to change to ensure particular populations with chronic health problems, such as ethnic minorities or socially disadvantaged children, receive equitable access to mental health services.

## CRIS: a sustainable resource for evaluating child health policy and service improvement

The CRIS system offers a sustainable resource for population-based analyses of linked patient level data to inform child mental health and acute hospital services and education services. Because CRIS uses data extracted from electronic record systems, it provides a powerful platform for continuous evaluation of local child health policy initiatives.^27^ The CRIS system provides an efficient, area-based resource for research, service planning and evaluation with patients followed up across the country. CRIS is being reproduced in other areas, potentially leading to a number of local areas having fine-grained information to better target local resources. However, there is still considerable work to be done. Health commissioners and other decision-makers at the local and national levels will need to develop sustainable means of implementing the knowledge which resources such as CRIS can deliver. This is essential if we wish to complete the Learning Health System cycle and use our informatics resources to drive healthcare improvement and innovation.^28^ Alongside this, public engagement, understanding and support are vital. If we want to adopt these systems further families, child health advocates, academics, clinicians and policy-makers will need to decide together how local linked resources are best safeguarded and used in commissioning services.

We hope in time that others will be encouraged to extend the CRIS model to link data for children across public services. By doing so, we hope to reduce the unmet need among vulnerable children and to move the discussions on from ‘not knowing’^29^ ^30^ to accurate and responsive information on which to base public health strategies for children and young people.
